# Protein Electrostatic Properties are Fine-Tuned Through Evolution

**DOI:** 10.21203/rs.3.rs-6471091/v1

**Published:** 2025-04-28

**Authors:** Mingzhe Shen, Guy W. Dayhoff, Jana Shen

**Affiliations:** † Department of Pharmaceutical Sciences, University of Maryland School of Pharmacy, Baltimore, MD 21201, U.S.A.; ‡ Joint first author

## Abstract

Protein ionization states provide electrostatic forces to modulate protein structure, stability, solubility, and function. Until now, predicting ionization states and understanding protein electrostatics have relied on structural information. Here we demonstrate that primary sequence alone enables remarkably accurate p*K*_a_ predictions through KaML-ESM, a model pretrained on a synthetic p*K*_a_ dataset that leverages evolutionary representations from large-scale protein language models ESMs. The KaML-ESM model achieves RMSEs approaching the experimental precision limit of ~0.5 pH units for Asp, Glu, His, and Lys residues, while reducing Cys prediction errors to 1.1 units – with further improvement expected as the training dataset expands. The state-of-the-art performance of KaML-ESM was further validated through external evaluations, including a proteome-wide analysis of protein p*K*_a_ values. Our results support the notation that protein sequence encodes not only structure and function but also electrostatic properties, which may have been co-optimized through evolution. Lastly, we provide KaML, a sequence-based end-to-end ML platform that enables researchers to map protein electrostatic landscapes, facilitating applications ranging from drug design and protein engineering to molecular simulations.

## Introduction

Protein structure and function are encoded in its amino acid sequence. Since ionization states play important roles in protein functions, we hypothesized that they can be predicted from the protein sequence alone. Emerging protein large language models (pLLMs) demonstrate powerful performance in predicting protein structures and functions through masked learning of protein sequences evolved over hundreds of millions of years.^1–4^ In a recent publication, the latest evolutionary scale model 3 (ESM3) was able to generate (without supervised learning) a fluorescent protein with a sequence identity of only 58% from known fluorescent proteins.^4^ This is because the representations emerging within the pLLMs reflect the biological structure and function of proteins and improve with scale, e.g., ESM3 is trained with 2.78 billion protein sequences.^4^

We posited that residue-level representations learned by pLLMs such as ESMs encode information about ionization states of protein sidechains and p*K*_a_ shifts that occur when residues transition from solution to protein environment, which are often large in magnitude for functional sites. To test this, we developed sequence-based p*K*_a_ prediction models, where per-token (i.e., residuespecific) embeddings extracted from specific layers of an ESM model were used as inputs to a multilayer perceptron (MLP; a feed-forward neural network with fully connected neurons) for predicting residue-specific p*K*_a_ values. The MLP is trained on the experimental p*K*_a_ database PKAD-3^5^ which is an expansion and refinement of the widely used PKAD-2 database.^6^ We named the new models KaML-ESMs, as the training protocol is derived from our most recent structure-based KaML (p***K***_**a**_ **M**achine **L**earning) models, especially KaML-CBTree which achieved the state-of-the-art (SOTA) prediction accuracies for all five amino acids, Asp, Glu, His, Lys, and Tyr.^5^

The sequence-based KaML-ESM models establish a new SOTA in p*K*_a_ predictions, pushing the accuracy boundary to near the experimental precision (about 0.5 pH units) for Asp, Glu, His and Lys, while reducing the average p*K*_a_ error of Cys to 1.1 pH units. External validation using newly curated experimental data confirms predictive performance. These results suggest that protein sequence encodes not only structure and function but also electrostatic properties, which may have been co-optimized through evolution. We developed an end-to-end p*K*_a_ sequence-based platform KaML and performed proteome-wide p*K*_a_ predictions for proteins identified in chemical proteomic experiments to further validate model performance and platform efficiency. We expect KaML to enable a wide range of applications, from drug design and protein engineering to molecular dynamics simulations.

## Results and Discussion

### ESM-learned representations can differentiate between identical amino acids with distinct p*K*_a_ shifts.

**Figure 1:**
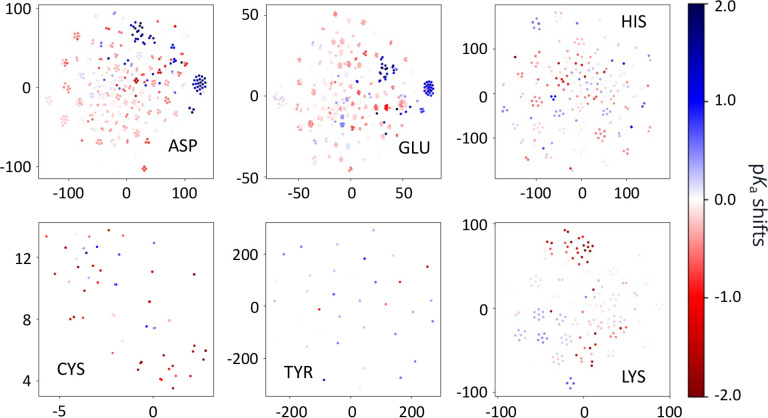
Residue-specific representations can differentiate between identical amino acids with distinct p*K*_a_ shifts. t-SNE visualization of the per-token embeddings (1280-digit) extracted from layer 31 of ESM2 650M for six titratable amino acids which have experimental p*K*_a_ values from the PKAD-3 database.^5^ Data points are colored according to the p*K*_a_ shifts relative to the solution values.

We first tested if the residue-specific representations extracted from the pLLM ESM2^3^ (trained with ~65 million unique sequences and 650 million parameters, see Supplemental methods) could differentiate between identical titratable amino acids exhibiting distinct p*K*_a_ shifts from the solution values. To do so, we employed t-distributed stochastic neighbor embedding (t-SNE) algorithm^7,8^ to generate two-dimensional visualization of pairwise similarities between identical amino acids with experimental p*K*_a_ values from the PKAD-3 database^5^ (Fig. 1). Significant positive p*K*_a_ shifts of Asp and Glu form the most prominent clusters. Considering that carboxylic acids with upshifted p*K*_a_’s are enriched in functional sites, this analysis confirms that residue-level evolutionary conservation and functional properties encoded by the ESM2 embeddings are indeed linked to p*K*_a_ shifts. Clustering of positive and negative p*K*_a_ shifts is also observed for His and Lys, while patterns appear less distinct for Cys and are absent for Tyr likely due to limited training data (60 Cys and 39 Tyr p*K*_a_ values).

### Pretraining combined with distinct acid and base models boosts the performance of KaML-ESM models for p*K*_a_ predictions.

Encouraged by the t-SNE analysis, we proceeded to build a KaML-ESM model, in which ESM serves as a foundation model to generate residue embeddings from protein sequences which are then fed to an MLP trained on the experimental p*K*_a_ shifts from the PKAD-3 database^5^ (Supplemental methods). Since PKAD-3 is small (1,167 p*K*_a_’s of 330 Asp, 382 Glu, 219 His, 60 Cys, 39 Tyr, and 137 Lys in 247 unique proteins), we conducted model pretraining using a synthetic dataset comprised of the p*K*_a_ shifts of 29,457 residues in 9,945 proteins predicted by the KaML-CBTree model, which demonstrated SOTA performance previously^5^ (details see Supplemental Methods and Fig. S1). The pretrained model was then fine-tuned on PKAD-3.^5^

Due to the distinctive mechanisms of p*K*_a_ shifts for acidic (Asp, Glu, Cys, Tyr) and basic (His and Lys) residues,^5^ we reasoned that training separate acid and base models is more appropriate. To evaluate the contributions from model pretraining (PT) and separation of acid/base models (AB), we trained and tested four KaML-ESM2 models: (1) no PT and no AB (baseline model); (2) PT only; (3) AB only; and (4) PT and AB. Compared to the baseline model, applying PT decreased the hold-out test root-mean-square error (RMSE) from 0.93 to 0.89, and applying AB decreased the test RMSE from 0.93 to 0.76 (Supplemental Table S1). Moreover, the combination of PT and AB demonstrates a synergistic effect, reducing RMSE from 0.93 to 0.73 (Supplemental Table S1). Therefore, the remainder of the work will focus on models trained using both strategies.

### Representation learning rates vary across amino acids.

Current pLLMs such as ESMs are built on the Transformer architecture,^9^ which processes inputs through a series of blocks that alternate self-attention with feed-forward connections.^1^ Consequently, pLLMs enable each residue to allocate “heightened attention” (increased attention weights) to important residues independently, regardless of their distances in the protein sequence. One earlier study analyzed pLLMs and found that different aspects of protein evolutionary features such as structures and functions are learned across different layers of the transformer, with deeper layers attending to residue contact relationships.^10^ Our recent development of structure-based KaML models demonstrated that protein p*K*_a_ values can be accurately predicted from the local structural environment.^5^ Considering that protein contact maps reflect the local environment, we asked if there is a particular ESM2 layer that offers the most accurate representation of protein ionization states. To test this, we extracted residue embeddings from the final 50% of layers (17–33) and evaluated their effectiveness by training dedicated models using each layer’s embeddings and examining the overall and amino acid-specific RMSEs. To reduce computational cost, layer evaluation was conducted without model pretraining unless otherwise noted.

**Figure 2:**
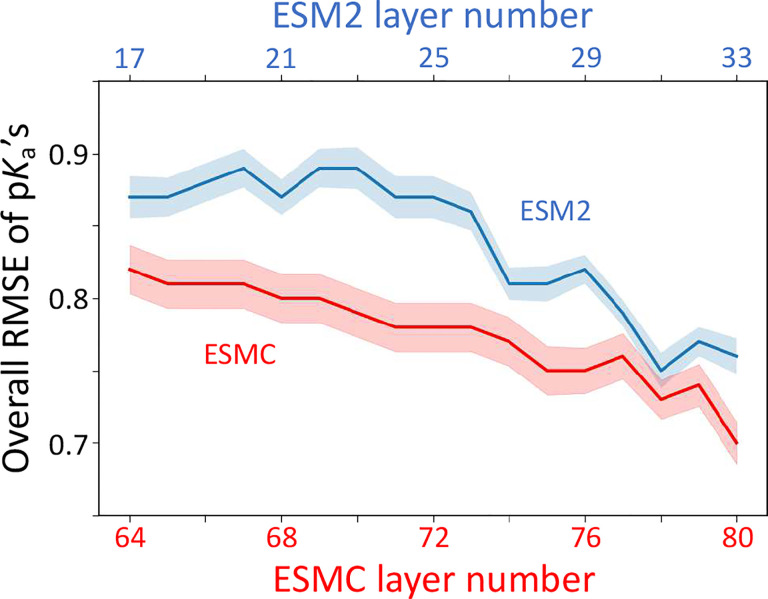
ESM2 and ESMC exhibit distinct representation learning patterns. Overall RMSEs of the p*K*_a_’s predicted by models trained with embeddings from specific transformer layers up to the final layer (33 for ESM2 and 80 for ESMC). The shaded regions represent the standard errors from 20 hold-out tests. Data for ESM2_650M and ESMC_6B are colored blue and red, respectively. No model pretraining was performed.

Interestingly, the overall test RMSE of the model does not decrease monotonically as learning progresses through the transformer layers; instead, it exhibits multiple local minima (Fig. 2, blue curve). This is due to the different rates of representation learning for different amino acids. The model RMSEs for Cys, His, Lys p*K*_a_’s decrease to the lowest value in the final layer (33), while the RMSEs for Asp, Glu, and Tyr reach minima at layers 31, 31, and 30, respectively (Supplemental Table S2). To confirm this pattern, we examined the last few layers by including model pretraining (Supplemental Table S2). The trend is similar, with layer 31 giving the lowest overall RMSE while amino acid-specific RMSEs reach minima at different layers. Since layer 31 embeddings yield the lowest overall test RMSE (0.68), we focus our subsequent discussion on this model and refer to it as KaML-ESM2.

### Influence of the ESM parameter scale and capacity of learning emergent structures.

We asked whether ESM’s parameter scale and architectural design influence its representation learning capabilities for protein ionization states. To address these questions, we trained KaML-ESM models using the embeddings from ESM2_15B.^3^ and the latest ESM Cambrian (ESMC, 6B parameters),^11^ which predicts emergent structures with significantly higher precision than ESM2 (even those with larger parameter scales) due to the use of a different architecture and orders of magnitude larger protein sequence space (2.78 billion sequences).^4,11^

For ESM2 15B, the models trained with the final four layers (45–48) give similar performances, with layer 47 (the second last) achieving the lowest RMSE of 0.73 (Supplemental Table S3). In contrast to ESM2 650M and ESM2 15B, the model trained with the final layer (layer 80) of ESMC gives the lowest RMSE of 0.70 (Fig. 2 and Supplemental Table S4). Interestingly, using the final 17 layers of ESMC, the overall RMSE steadily decreases with progressively deeper layers (Supplemental Table S4), suggesting that the model’s capacity to learn protein electrostatic properties may not have reached saturation. The overall RMSEs of KaML-ESMC are consistently lower than the corresponding RMSEs of KaML-ESM2 (with 640M or 15B parameters), suggesting that the enhanced capacity of learning emergent structures^4,11^ plays a more important role in accurate prediction of electrostatic properties than raw parameter scale. It is also noteworthy that the amino acid-specific RMSEs decrease steadily toward the final layer, suggesting that the representation learning rates across amino acids are more uniform compared to ESM2. Since the best KaML-ESM2_15B model gives a higher RMSE, we drop the model in the following discussion. We then retrained KaML-ESMC using the representations of the final layer (80) by including pretraining. We refer to it as KaML-ESMC hereafter.

### KaML-ESM2a and KaML-ESMCb establish a new SOTA benchmark for predicting p*K*_a_’s and protonation states.

We compared the RMSE, Pearson’s correlation co-efficient (PCC), and maximum error (MAXE) of the predicted p*K*_a_’s of acidic and basic residues by KaML-ESMs and structure-based KaML-CBTree and the empirical PROPKA3 method (Table 1). For clarity, we added a suffix a or b to denote the model type, i.e., KaML-ESM2a/KaML-ESMCa for acidic and KaML-ESM2b/KaML-ESMCb for basic p*K*_a_ predictions.

In identical 20 hold-out tests, both acid and base KaML models outperform the previous SOTA ML p*K*_a_ predictor KaML-CBTree, which substantially surpasses the widely-used PROPKA3 method (Table 1). Interestingly, KaML-ESM2 and KaML-ESMC demonstrate complementary strengths: KaML-ESM2a excels at predicting acidic residue p*K*_a_’s (RMSE=0.67; PCC=0.91), while KaML-ESMCb achieves superior performance for basic residues (RMSE=0.57; PCC=0.96).

We also examined the protonation-state prediction metrics, precision, recall, and critical error rates. Following our previous work,^5^ the continuous p*K*_a_ values are discretized into three classes based on the protonation probability (Prob) at pH 7: protonated (Prob >0.75, p*K*_a_ <6.52), deprotonated (Prob <0.25, p*K*_a_ >7.48), and titrating (0.25≤ Prob ≤0.75, 6.52≤ p*K*_a_ ≤7.48). According to all classification metrics, both KaML-ESMs outperform KaML-CBTree and PROPKA3 (Table 1). Consistent with the p*K*_a_ regression metrics, KaML-ESM2a delivers the highest recall, precision, and lowest critical error rates (CERs) when classifying protonation states of acidic residues, while KaML-ESMCb provides the best classification metrics for basic residues (Table 1).

When evaluating individual amino acid p*K*_a_ and protonation state predictions, KaML-ESM2a establishes a new SOTA for Asp, Glu, and Cys, while KaML-ESMCb a new SOTA for His and Lys (Table 2, Supplemental Fig. S4 and Fig. S5). An exception is Tyr, for which KaML-CBTree remains the SOTA performer, which is unsurprising given the decision tree’s effectiveness when trained on the extremely small dataset of just 39 Tyr p*K*_a_’S.

### KaML-ESMs offer the most significant improvement for predicting Cys and His p*K*_a_’s and protonation states.

The most significant improvement over the previous SOTA KaML-CBTree (KaML-CBT) is for Cys and His. Comparing KaML-ESM2a and KaML-CBTa, the RMSE of Cys p*K*_a_’s is reduced by 0.4 units and the CER is reduced by 25% (Table 2). KaML-ESM2a achieves the precision and recall of 89% and 88% in predicting Cys^−^, as compared to 73% and 76% by KaML-CBTa, respectively. This level of performance in predicting deprotonated cysteines, which are highly nucleophilic and frequent linkage sites for targeted covalent inhibitors,^13^ positions KaML-ESM2a as a valuable tool for rational covalent drug design.

The second most significant improvement is for His. The solution p*K*_a_ of His is ~6.5,^14,15^ which is close to the cytosolic pH 7.1. This means that any small errors in p*K*_a_ prediction may lead to a critical error (predicting protonated as deprotonated state or vice versa). Along with a 0.24-unit decrease in RMSE when comparing KaML-ESMCb to KaML-CBTb, the CER is reduced by threefold and the recall for His^+^ increased from 0.37 to 0.90 with the precision of 0.92 (Table 2 and Supplemental Fig. S4 and Fig. S5). This remarkable improvement suggests that KaML-ESMCb can be used to improve fidelity of molecular dynamics (MD) simulations, which typically set histidines in the neutral state.

### KaML-ESM predictions for Asp, Glu, His, and Lys approach the experimental precision.

An earlier study that analyzed NMR titration data from different laboratories suggested that the minimum average error of p*K*_a_ estimates is roughly 0.5 units.^16^ Using this knowledge as a guide and noting the convergence in RMSE (~0.6) for Glu between the predictions by KaML-ESM2a, KaML-CBTa (and KaML-ESMa), we suggest that the models have reached an accuracy threshold approaching the experimental measurement uncertainty. Similarly, KaML-ESM2a appears to approach the performance ceiling for Asp, while KaML-ESMCb appears to approach the performance ceiling for His and Lys. Hereafter, we refer to the combined KaML-ESM2a and ESMCb model as KaML-ESM.

### External evaluation of KaML-ESM confirms the SOTA performance.

To provide a production KaML-ESM model to the community, the models were retrained using the complete dataset (Supplemental methods). The production KaML-ESM was further evaluated on a newly collected experimental dataset composed of p*K*_a_ values of 55 residues (39 His, 3 Cys, and 13 Lys) from 16 proteins, which are not in PKAD-3^5,6^ (Supplemental Fig. S6). Examining individual amino acids, KaML-ESM gives RMSE of 0.52 for His, 0.60 for Cys, and 0.47 for Lys, as compared to the respective RMSEs of 0.57, 0.87, 0.97 with KaML-CBTree. The enhanced performance stems from eliminating KaML-CBTree’s systematic tendency to overestimate p*K*_a_ downshifts of Cys and Lys residues. Lastly, no critical errors are found in the KaML-ESM predictions.

### Developing the KaML platform for p*K*a predictions and visualization.

**Figure 3:**
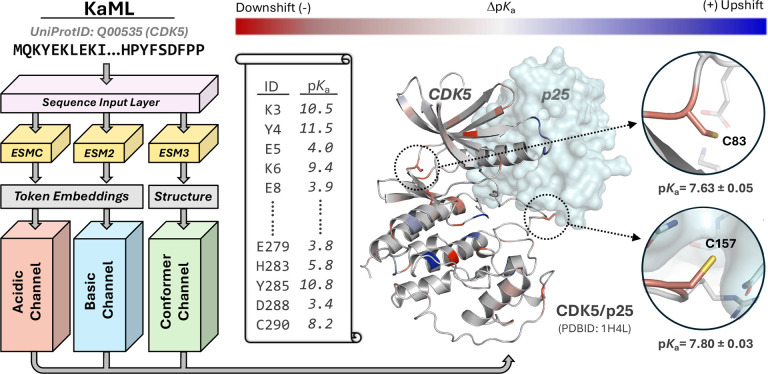
Architecture of the KaML platform and illustration of p*K*_a_ predictions. KaML accepts a user-provided protein sequence through the sequence input layer. The pLLMs, ESMC and ESM2, generate token embeddings, while a third pLLM, ESM3, predicts a three-dimensional structure if not provided. Embeddings from ESM2 and ESMC feed the acidic and basic channels (ensemble of 200 MLPs), respectively. Optionally, the ESM3-derived or user-provided structure may be processed by the conformer channel for conformational state-dependent predictions (e.g., by KaMLs-CBTree). Outputs from the acidic and basic channels are combined to yield predicted p*K*_a_’S, shifts relative to solution values, standard errors, and conformational state-dependent p*K*_a_’s (optional). A vertical scroll illustrates the output for CDK5. A cartoon representation of CDK5 is given, with the binding partner P25 (not included in the prediction) shown in the surface view. Residues with up- and down-shifted p*K*_a_’s are colored in blue and red, respectively. Two cysteines are highlighted and discussed in the main text.

To provide an accessible tool for the scientific community, we developed a sequence-based p*K*_a_ prediction platform, utilizing ESM2^3^ and ESMC^11^ as foundation models for downstream sequence-based p*K*_a_ predictions and the most recent ESM3^4^ to generate protein structures for visualization and optional structure-based p*K*_a_ predictions (Fig. 3 left, Supplemental Methods). To make KaML-ESM broadly usable, we provide both a command-line interface and an online browser-based GUI (https://kaml.computchem.org) that support input via protein sequence, UniProt ID, PDB ID, or user-provided PDB file, along with an ESM Forge API token to initiate predictions (Supplemental Methods and Fig. S9).

Fig. 3 illustrates the p*K*_a_ prediction results for an example protein, cyclin-dependent kinase 5 (CDK5), which regulates the mammalian central nervous system.^17^ The prediction process takes about 35 seconds on the command line or web interface. KaML-ESM predicts that Cys83 and Cys157 have down-shifted p*K*_a_ values, i.e., both are highly reactive, consistent with the finding that they are modified by S-nitrosylation events in neurodevelopmental and neurodegenerative processes.^18^ Our previous work showed that highly reactive cysteines adjacent to binding pockets can serve as covalent linkage sites for targeted covalent inhibitors.^13,19^ Consistent with its reactivity, Cys157 has been identified as ligandable in chemoproteomic experiments,^20–22^ which offers an exciting opportunity for disrupting the interface between CDK5 and p25, as aberrant formation of this complex leads to CDK5 hyperactivation, contributing to tau hyperphosphorylation and neurodegeneration.^17,23^

### Proteome-wide predictions further validate the accuracy for Asp/Glu/His/Lys while highlighting improvement opportunities for Cys/Tyr.

**Figure 4:**
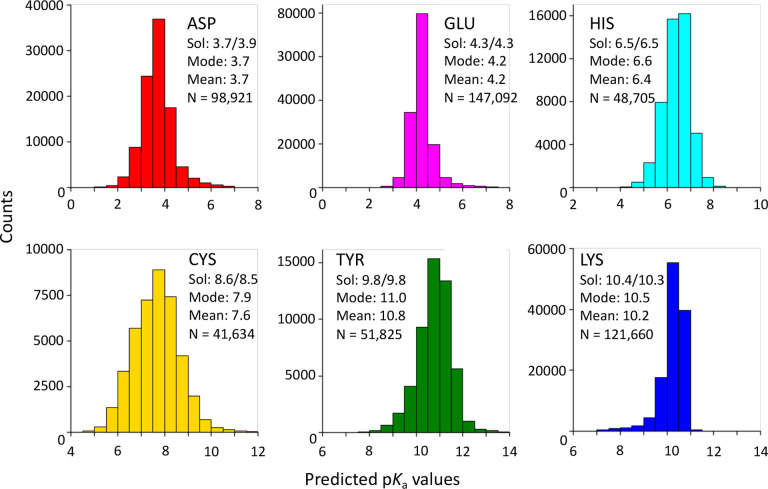
Proteome-wide p*K*_a_ predictions by KaML-ESM. Histograms of the predicted p*K*_a_’s of Asp, Glu, His, Cys, Tyr, and Lys in proteins identified by the chemical-proteomic experiments.^20–22,24–30^ Sol: p*K*_a_ in the model tripeptide (GXG)^14^ or penta-peptide (AAXAA);^15^ Mode: most probable p*K*_a_; Mean: average p*K*_a_; N: total number of residues. The dataset contains 509,837 residues from proteins expressed by 3,892 unique genes with a sequence length < 1022.

To further validate the model performance and demonstrate the high-throughput capability of KaML-ESM, we made sequence-based p*K*_a_ predictions for proteins identified in chemoproteomic activity-based protein profiling (ABPP) experiments across various cell lines.^20–22,24–30^ A total of 509,837 p*K*_a_ values of Asp, Glu, His, Cys, Tyr, and Lys residues in proteins expressed by 3,892 unique genes with sequence length < 1022 were predicted (Fig. 4). Note, the majority of these proteins do not have experimental structures. Remarkably, the p*K*_a_ ranges of Asp, Glu, His, and Lys reflect the experimental distributions of PKAD-3^5^ and the mode/mean p*K*_a_ values align within 0.1 units from their solution p*K*_a_ values (Fig. 4), despite this not being an explicit model constraint – supporting our hypothesis that KaML-ESM predictions approach experimental precision.

In contrast, compared to the solution values, the mode/mean of the predicted p*K*_a_’s of Cys is lower by 0.6–0.9 units, while that of Tyr is higher by 1.0–1.2 units. These deviations are consistent with the higher prediction errors for Cys and Tyr (RMSEs of 1.1 and 1.2, respectively) and suggest the presence of systematic errors, which are attributed to the limited training data (60 Cys and 39 Tyr p*K*_a_’S).

### Recent related work based on pLLMs.

Prior to submission, we became aware of an ESM-derived p*K*_a_ prediction model pKAML,^31^ which utilizes the concatenated vectors comprising the embeddings from a pLLM (e.g., ESM2) and the predicted protein and peptide isoelectric points. pKAML differs from KaML-ESM in many aspects. pKAML is a combined acid and base model directly trained (i.e., no pretraining) on experimental p*K*_a_ shifts from a subset of the PKAD-2 database^6^ (significantly smaller than PKAD-3^5^ used in this work) and evaluated on a single hold-out test. Among the evaluated pLLMs, pKAML based on ESM2 35M gives the best performance, achieving an overall RMSE of 0.9 in the hold-out test. In comparison, KaML-ESM gives an overall RMSE of 0.65±0.10 in 20 hold-out tests. When evaluated on our external test data, pKAML gives an RMSE of 0.67, compared to the RMSE of 0.49 given by KaML-ESM (Supplemental Fig. S6).

## Concluding Discussion

Protein ionization states provide electrostatic forces to modulate protein structure, stability, solubility, and function. In the past, prediction and interpretation of ionization states have relied on structure-based approaches, including physics-based calculations,^32,33^ empirical methods,^12^ and ML models.^5,32,34–36^ Our work establishes that primary sequence alone enables remarkably accurate p*K*_a_ predictions. The KaML-ESM model achieves RMSEs approaching experimental precision limits (~0.5 pH units) for Asp, Glu, His, and Lys residues, while reducing Cys prediction errors to 1.1 units – with further improvement expected as the training dataset expands. These results support the notion that protein sequence encodes not only structure and function but also precise electrostatic properties, which may have been co-optimized through evolution.

Beyond improving p*K*_a_ prediction accuracy for Cys and Tyr through the use of larger training dataset, the KaML platform can be refined and expanded. For example, our analysis revealed distinct representation learning patterns across amino acids. While learning saturates at layer 31 for carboxylic acids, the RMSE for Cys continues to decrease, reaching 1.0 in the ESM2 model’s final layer (33). This suggests the potential to develop more refined models that leverage amino acid-specific layer embeddings for improved predictive performance. While KaML-CBTree is currently used to predict conformational state-dependent p*K*_a_’s incorporating sequence information and making use of other architectures may improve model performance. Finally, we envisage the integration of KaML with constant pH MD simulation^33^ to model the dynamic interplay between protonation state changes and conformational transitions critical to biological functions. Such an integrated approach would further advance our understanding of how electrostatic remodeling drives protein functions, e.g., in proton-coupled gating of ion channels and activation of membrane transporters.

## Figures and Tables

**Table 1:** Comparison of sequence-based KaML-ESMs against structure-based KaML-CBTree and empirical PROPKA3 for acid and base p*K*_a_ and protonation state prediction^a^

	KaML-ESM2		KaML-ESMC		KaML-CBTree		PROPKA3	
	KaML-ESM2a	KaML-ESMb	KaML-ESMCa	KaML-ESMCb	KaML-CBTa	KaML-CBTb	acid	base
**RMSE**	**0.67 ± 0.03**	0.68 ± 0.03	0.71 ± 0.04	**0.57 ± 0.02**	0.76 ± 0.03	0.79 ± 0.02	1.28 ± 0.03	0.96 ± 0.04
**PCC**	**0.91 ± 0.01**	0.94 ± 0.01	0.90 ± 0.01	**0.96 ± 0.01**	0.88 ± 0.01	0.92 ± 0.01	0.74 ± 0.01	0.90 ± 0.01
**MAXE**	**3.01 ± 0.17**	2.17 ± 0.16	3.21 ± 0.24	**1.93 ± 0.16**	3.17 ± 0.14	2.60 ± 0.16	3.72 ± 0.06	5.04 ± 0.10
**Classification of protonation states at pH 7** ^b^								
**Pre (prot)**	**0.94**	0.98	**0.94**	**0.99**	0.91	**0.99**	0.66	0.97
**Rec (prot)**	**0.93**	0.98	0.83	**0.99**	0.82	0.97	0.78	0.88
**Pre (dep)**	**0.99**	0.97	**0.99**	**0.99**	**0.99**	0.95	0.98	0.97
**Rec (dep)**	**1.00**	0.97	**1.00**	**0.99**	0.99	**0.99**	0.97	0.85
**CER** ^c^	**20/2062**	13/545	38/2081	**5/572**	34/2099	12/536	90/2055	53/618

a The averages and standard errors from 20 hold-out tests are shown. The metrics of KaML-CBTree^5^ and PROPKA3^12^ are taken from Ref.^5^ The best metrics are highlighted in bold font. The p*K*_a_’s for acidic (Asp, Glu, Cys, and Tyr) and basic residues (His and Lys) are predicted by acid and base KaMLs, respectively, while a single PROPKA3 model makes prediction for all residue types.

b Prediction is based on the probability of protonation given a predicted p*K*_a_ (see main text).

c Critical error rate (CER) refers to the percentage of predictions misclassifying protonated as deprotonated or vice versa. Precision (Pre) and recall (Rec) were calculated for protonated (prot) and deprotonated (dep) states after accumulating the predictions from all 20 holdout test sets.

**Table 2:** Comparison of sequence-based KaML-ESMs against structure-based KaML-CBTree and empirical PROPKA3 for amino acid p*K*_a_ and protonation state prediction^a^

	KaML-ESM2a		KaML-ESMCa		KaML-CBTa		PROPKA3	
	RMSE	CER	RMSE	CER	RMSE	CER	RMSE	CER
**Asp**	**0.61 ± 0.04**	3/904	0.64 ± 0.05	10/913	0.75 ± 0.04	13/916	1.12 ± 0.04	31/907
**Glu**	**0.58 ± 0.04**	5/1056	0.61 ± 0.03	17/1066	0.60 ± 0.02	5/1076	1.02 ± 0.05	21/1045
**Cys**	**1.11 ± 0.09**	9/63	1.25 ± 0.13	8/63	1.50 ± 0.13	13/68	3.58 ± 0.18	35/66
**Tyr**	1.54 ± 0.16	–	1.45 ± 0.16	–	**1.24 ± 0.19**	–	1.67 ± 0.18	–
	KaML-ESM2b		KaML-ESMCb		KaML-CBTb		PROPKA3	
	RMSE	CER	RMSE	CER	RMSE	CER	RMSE	CER
**His**	0.68 ± 0.03	6/220	**0.61 ± 0.03**	3/251	0.85 ± 0.03	11/209	1.03 ± 0.06	47/303
**Lys**	0.69 ± 0.07	7/325	**0.50 ± 0.03**	2/321	0.70 ± 0.05	1/325	0.80 ± 0.05	6/315

a The averages and standard errors from 20 hold-out tests are shown.

Metrics for KaML-CBTree^5^ and PROPKA3^12^ are taken from Ref.^5^ The lowest RMSEs are highlighted in bold font. CER of Tyr is not calculated due to the extremely small test sets (3 Tyr^−^).

## Data Availability

The PKAD-3 database is freely searchable and downloadable at https://database.computchem.org/pkad-3. The ABPP dataset used in this work is collected from Refs.^20–22,24–30^
